# Integrated Assessment of Heavy Metal Pollution in the Surface Sediments of the Laizhou Bay and the Coastal Waters of the Zhangzi Island, China: Comparison among Typical Marine Sediment Quality Indices

**DOI:** 10.1371/journal.pone.0094145

**Published:** 2014-04-07

**Authors:** Wen Zhuang, Xuelu Gao

**Affiliations:** 1 Key Laboratory of Coastal Environmental Processes and Ecological Remediation, Yantai Institute of Coastal Zone Research, Chinese Academy of Sciences, Yantai, Shandong, China; 2 College of City and Architecture Engineering, Zaozhuang University, Zaozhuang, Shandong, China; 3 University of Chinese Academy of Sciences, Beijing, China; University of California, Merced, United States of America

## Abstract

The total concentrations and chemical forms of heavy metals (Cd, Cr, Cu, Ni, Pb and Zn) in the surface sediments of the Laizhou Bay and the surrounding marine area of the Zhangzi Island (hereafter referred to as Zhangzi Island for short) were obtained and multiple indices and guidelines were applied to assess their contamination and ecological risks. The sedimentary conditions were fine in both of the two studied areas according to the marine sediment quality of China. Whereas the probable effects level guideline suggested that Ni might cause adverse biological effects to occur frequently in some sites. All indices used suggested that Cd posed the highest environmental risk in both the Laizhou Bay and the Zhangzi Island, though Cd may unlikely be harmful to human and ecological health due to the very low total concentrations. The enrichment factor (EF) showed that a substantial portion of Cr was delivered from anthropogenic sources, whereas the risk assessment code (RAC) indicated that most Cr was in an inactive state that it may not have any adverse effect either. Moreover, the results of EF and geoaccumulation index were consistent with the trend of the total metal concentrations except for Cd, while the results of RAC and potential ecological risk factor did not follow the same trend of their corresponding total metal concentrations. We also evaluated the effects of using different indices to assess the environmental impact of these heavy metals.

## Introduction

Heavy metals in ecosystems have received extensive attention because they are toxic, non-biodegradable in the environment and easy to accumulate and magnify in organisms. Concentrations of heavy metals in aquatic ecosystems have increased considerably due to the inputs of industrial waste, sewage runoff and agriculture discharges [1], [2]. In other words, heavy metal pollution may likely go with the rapid economic development [3], [4]. The measurements of heavy metals only in the water and in the suspended material are not conclusive due to water discharge fluctuations and low resident time [5]. With a combined action of adsorption, hydrolysis and co-precipitation, only a small part of free metal ions stay dissolved in water, and a large quantity of them get deposited in the sediments [6]. However, when environmental conditions change, sediments may transform from the main sink of heavy metals to sources of them for the overlying waters [1], [7]. Therefore the contents of heavy metals in sediments are often monitored to provide basic information for environmental risk assessment [8], [9].

In recent decades, various risk assessment indices have been applied to evaluate the environmental risks of metals in marine sediments. Caeiro et al. [10] classified them into three types: contamination indices, background enrichment indices and ecological risk indices. In the present study contamination indices and background enrichment indices were collectively called contamination indices. To assess the metal contamination, the geoaccumulation index (*I*_geo_) [5], [11] and the enrichment factor (EF) [3], [12] are often used. Meanwhile, the risk assessment code (RAC) [13] and the potential ecological risk index (ER) [14] are very popular indices in evaluating the ecological risk posed by heavy metals in sediments. Therefore, these four indices were employed to assess the contamination and ecological risks of the six selected metals (i.e., Cd, Cr, Cu, Ni, Pb and Zn) in the surface sediments of the Laizhou Bay and the coastal waters of the Zhangzi Island (hereafter also referred to as Zhangzi Island for short), China. Numerous sediment quality guidelines (SQGs) also have been developed to deal with environmental concerns. Marine Sediment Quality of China (GB 18668-2002) [9] is one of the SQGs usually used as a general measure of marine sediment contamination in China, so this guideline was chosen in this study. TEL (threshold effects level) and PEL (probable effects level), which were proved to be effective sediment quality guidelines [15], [16], were also used in this study. Many studies have shown that different conclusions or even contradictory conclusions may be drawn by using different risk assessment methods for the same sample or for different elements within the same sample [10], [13], [17]. Therefore, the relationships among the four index methods and the total concentrations of metals were explored to find out their differences in the environmental risk assessment of heavy metals. Since all the four indices are very popular in evaluating the environmental risks posed by heavy metals in sediments all over the world, we hope to provide useful information about these indices for other researchers to refer to when they carry out similar studies.

To sum up, the purposes of this study are i) to quantify and explain the spatial distribution and fractionations of six heavy metals (Cd, Cr, Cu, Ni, Pb and Zn) in the surface sediments of the Laizhou Bay and the Zhangzi Island; ii) to explore the degree of contamination and the potential ecological risks of these heavy metals to the environment; and iii) to investigate the differences among the used risk assessment indices and the SQGs in the environmental risk assessment of heavy metals.

## Materials and Methods

### Ethics statement

**Figure 1:**
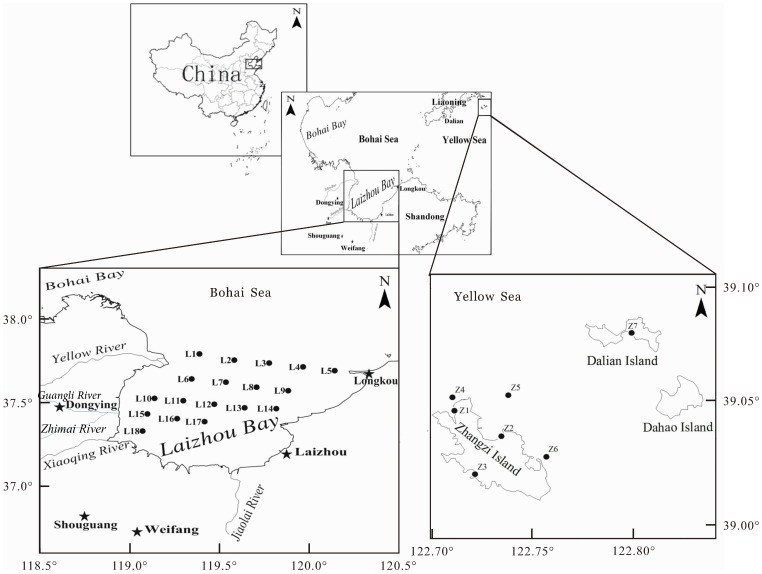
Location of sampling sites in the Laizhou Bay and the coastal Zhangzi Island.

This study did not involve endangered or protected species and no specific permissions were required for these locations/activities in this study. The specific locations of the present study were shown in Fig. 1.

### Study area

The Laizhou Bay (area–7000 km^2^, coastline length–320 km, mean depth <10 m, max. depth–18 m) lies in the southern part of the Bohai Sea, accounting for up to 10% of the total area (Fig. 1). It is a semi-closed shallow area with relatively flat seafloor which is formed by the accumulation of riverine suspended matters. There are more than a dozen of rivers running into the Laizhou Bay, among which the Yellow River and the Xiaoqinghe River influence the Laizhou Bay most. Riverine sediment load of the Yellow River and the Xiaoqinghe River began to decrease from the second half of the 20th century, whereas the amount of the contaminants brought by them increased with years [18], [19], [20]. From the western coast to the eastern coast of the Laizhou Bay, there in turn have Dongying Port, Yangjiaogou Port, Weifang Port and Longkou Port which are important ports in Shandong Province. Due to the abundant seawater resources and underground brine resources in the coastal Laizhou Bay, one of the biggest chemical industrial bases in the world called Weifang Binhai Economic Development Zone is located along its southwestern coast. More than 400 chemical enterprises are located nearby and large amounts of non-purified or insufficiently purified wastewaters are discharged into the Laizhou Bay. The Laizhou Bay can be characterized as a region surrounded by areas with high population growth and rapid economic development in China. The Laizhou Bay used to be one of the most important spawning and breeding grounds for many marine organisms in China. The rapid economic development has brought serious ecological damages to the Laizhou Bay, and fishery resources in the Laizhou Bay are gradually disappearing. The overall sharp decrease of fishery resources and unpredictable nature of the sediment highlight the necessity of the environmental risk assessments of pollutants, especially the heavy metals possessing high affinities for sedimentary materials.

The Zhangzi Island (coastline length–60 km) is located in a national first-class clean sea area in the northern North Yellow Sea which is ∼100 km away from Dalian City, Liaoning Province (Fig. 1). The offshore area of the Zhangzi Island is the largest aquaculture base for choice rare seafood in China, which produces conchs, sea cucumbers, scallops, abalones, sea urchins and so on. The only national original seed field for *Patinopecten yessoensis* is located in this area. Fodder-feeding has been banned from seafood farming for many years in this area, and the cultivation of crops on the island is also prohibited. It is thought that natural environment in the Zhangzi Island is generally better than other typical Chinese coastal seas such as the Laizhou Bay. However, sewage (residual feeds, excrement and suspended particles, etc.) discharged during the process of raising seedlings and the frequent operation of ships may result in the deterioration of the local waters and sediment qualities.

### Sampling

In this study, a total of 18 surface sediment samples were collected in the Laizhou Bay in October 2011 and 7 surface sediment samples were collected in the Zhangzi Island in November 2011 (Fig. 1). In the Laizhou Bay, sampling sites L1 and L6 were near to the new and old mouths of the Yellow River, respectively; site L18 was near to the estuary of the Xiaoqinghe River. In the Zhangzi Island, the sampling sites were chosen stochastically. Three sites were located in the intertidal zone (Z1–Z3); three were in the coastal waters (Z4–Z6), among which Z4 and Z5 were located in the mariculture areas where sea cucumbers and scallops were farmed, respectively. Site Z7 was in the intertidal zone of an outer island called the Dalian Island. Surface sediment samples (∼0–5 cm) were collected by a stainless steel grab sampler and/or a plastic spatula, and were placed in acid-rinsed polyethylene bags. They were transported to the laboratory in a cooler box with ice packs and stored at 4 °C until further treatment.

### Analytical methods

The information about the fractionations of metals in the surface sediments was obtained by a sequential extraction procedure reported by Rauret et al. [21]. The four operationally defined geochemical fractions which were separated under this scheme are acid soluble, reducible, oxidizable and residual. The detailed sequential extraction protocol used in this study has been described elsewhere [22].

Previous experiments have shown that sample drying could alter the solid phase distribution of trace elements [23], [24]. Furthermore, the elemental concentrations in sediments are highly dependent on the grain size [25], [26]. So a drying and grinding treatment could potentially alter the extractability of elements [27]. For the reasons above, wet and unground sediments were used for the sequential extraction procedure in this study to reduce errors.

The mixture of concentrated HF, HNO_3_ and HClO_4_ (5∶2∶1) [22] was used to digest five randomly selected residues instead of the so-called pseudototal digestion with aqua regia used by Rauret et al. [21]. The total metal concentrations in all samples were obtained by the same method used to get the metal concentrations in the residual fraction. The concentrations of metals in residual fractions were estimated by subtracting the metal concentrations obtained in the first three steps of sequential extraction from the total metal concentrations. The sum of the measured values of the four geochemical fractions accounted for 85–110% of the values from the total digestion experiment.

Inductively coupled plasma mass spectrometry (PerkinElmer Elan DRC II) was applied in this work for the determination of Cd, Cr, Cu, Pb, Ni and Zn. In addition, the concentration of Al was analyzed by inductively coupled plasma optical emission spectrometer (PerkinElmer Optima 7000 DV) and the enrichment factor for each element was calculated.

The total organic carbon (TOC) in sediments was obtained by determining the total carbon using an Elementar vario MACRO cube CHNS analyzer after removing the inorganic carbon with 1 M HCl. The substance concentrations of sediments were expressed on the dry weight basis based on the results of moisture contents, which were determined gravimetrically by comparing the weight differences before and after heating an aliquot of sediment at 105 °C until constant weight was obtained. The grain size of samples was analyzed by a Malvern Mastersizer 2000 laser diffractometer capable of analyzing particle sizes between 0.02 and 2000 μm. The percentages of the following three groups of grain sizes were determined: <4 μm (clay), 4–63 μm (silt), and >63 μm (sand) [3], [21].

### Quality control

The analytical data quality was guaranteed through the implementation of laboratory quality assurance and quality control methods, including the use of standard operating procedures, calibration with standards, analysis of reagent blanks, and analysis of replicates. The precision of the analytical procedures was tested by recovery measurements on the Chinese national geostandard samples (GBW-07333 and GBW-07314). The results were consistent with the reference values, and the differences were all within 10%. The precision of the analytical procedures, expressed as the relative standard deviation (RSD), ranged from 5% to 10%. The precision of the analysis of standard solution was better than 5%. All analyses were carried out in duplicate, and the results were expressed as the mean.

### Assessment of sediment contamination and ecological risks

In this study, four different indices were used to assess the degree of heavy metal contamination and ecological risks in the surface sediments of the Laizhou Bay and the Zhangzi Island. For the comparison purpose, the average upper continental crust (UCC) values [29] were chosen as the reference background values in all of the following related indices (Table 1).

**Table 1:** The metal guideline values of two different criteria used to distinguish marine sediment quality and the average upper continental crust (UCC) values.

Sediment quality guidelines	Cd	Cr	Cu	Ni	Pb	Zn	Reference
Class I upper limit	0.5	80	35		60	150	[9]
Class II upper limit	1.5	150	100		130	350	[9]
Class III upper limit	5	270	200		250	600	[9]
TEL guideline	0.68	52.3	18.7	15.9	30.2	124	[28]
PEL guideline	4.2	160	108	42.8	112	271	[28]
UCC	0.098	35	25	20	20	71	[29]

Content unit is μg g^−1^ dry weight for all elements.

#### 1. Enrichment factor (EF)

EF is a useful contamination index in determining the degree of anthropogenic heavy metal pollution. The EF for each element was calculated to evaluate anthropogenic influences on heavy metals in sediments using the following formula [3]:
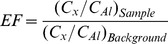



Where *C*_x_ and *C*_Al_ denote the concentrations of element x and Al in the samples and in UCC, respectively. In this study, Al was used as the reference element for geochemical normalization, because it represents the quantity of aluminosilicates which is generally the predominant carrier phase for metals in coastal sediments and its natural concentration tends to be uniform [30]. According to EF values, each sample falls into one of the seven tiers: i) EF<1 indicates no enrichment; ii) 1<EF<3 is minor enrichment; iii) 3<EF<5 is moderate enrichment; iv) 5<EF<10 is moderately severe enrichment; v) 10<EF<25 is severe enrichment; vi) 25<EF<50 is very severe enrichment; and vii) EF>50 is extremely severe enrichment [12].

#### 2. Geoaccumulation index (*I*_geo_)

*I*_geo_ is also a contamination index which is defined by the following equation:
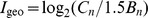



Where *C*_n_ is the measured concentration of metal n; *B*_n_ is the geochemical background concentration of metal n. Correction index 1.5 is usually used to characterize the sedimentary and geological characteristics of rocks and other effects [5]. The geoaccumulation index consists of seven classes: *I*_geo_≤0 (Class 0, practically uncontaminated); 0<*I*_geo_≤1 (Class 1, uncontaminated to moderately contaminated); 1<*I*_geo_≤2 (Class 2, moderately contaminated); 2<*I*_geo_≤3 (Class 3, moderately to heavily contaminated); 3<*I*_geo_≤4 (Class 4, heavily contaminated); 4<*I*_geo_≤5 (Class 5, heavily to extremely contaminated); 5<*I*_geo_ (Class 6, extremely contaminated) [31].

#### 3. Risk assessment code (RAC)

RAC which was originally developed by Perin et al [32] is widely used in ecological risk assessments of heavy metals in sediments. RAC is defined as:
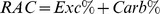



Exc% and Carb% are percentages of metals in exchangeable and carbonate fractions (i.e., acid soluble fractions in the present study). According to RAC values, each sample falls into one of the five tiers: i) RAC≤1% (no risk); ii) 1%<RAC≤10% (low risk); iii) 10%<RAC≤30% (medium risk); iv) 30%<RAC≤50% (high risk); v) 50%<RAC (very high risk).

#### 4. Potential ecological risk factor (ER)

ER was originally developed by Hakanson [33] and is also an index widely used in ecological risk assessments of heavy metals in sediments. According to this methodology, the potential ecological risk index is defined as:
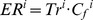


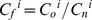



ER^i^ is the potential ecological risk factor for a given element i; Tr^i^ is the toxic-response factor for element i (e.g., Cd = 30, Cu = Pb = Ni = 5, Cr = 2, Zn = 1); *C*_f_^i^, *C*_o_^i^ and *C*_n_^i^ are the contamination factor, the concentration in the sediment and the background reference level for element i, respectively. According to Hakanson [33] the following tiers are used for the ER^i^ value: i) ER≤40 (low risk); ii) 40<ER≤80 (moderate risk); iii) 80<ER≤160 (considerable risk); iv) 160<ER≤320 (high risk); v) 320<ER (very high risk).

### Sediment quality guidelines

Numerous sediment quality guidelines (SQGs) have been developed to deal with environmental concerns, and two of them were chosen to assess the contamination extent of individual metals in the surface sediments of the Laizhou Bay and the Zhangzi Island (Table 1).

The marine sediment quality of China (GB18668-2002) [9] has defined three grades of marine sediments, in which the contents of five metals (i.e., Cd, Cr, Cu, Pb and Zn) are regarded as parameters used to classify marine sediment quality. According to this criterion, three classes are identified: i) mariculture, nature reserve, endangered species reserve, and leisure activities are suitable; ii) industry and tourism site can be established; iii) only used for harbor.

Threshold effects level (TEL) and probable effects level (PEL) are also sediment quality guidelines which are widely used [28]. TEL is the concentration below which adverse biological effects rarely occur; PEL is the concentration above which adverse biological effects frequently occur.

Based on the fact that heavy metals occur in sediments as complex mixtures, the mean PEL quotient method has been applied to determine the possible biological effect of combined toxicant groups by calculating the mean quotients for a large range of contaminants using the following formula [34]:
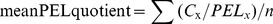



Where C_x_ is the sediment concentration of component x, PEL_x_ is the PEL for compound x and n is the sum of components. Based on the analyses of matching chemical and toxicity data from over 1000 sediment samples from the USA estuaries, the mean PEL quotients of <0.1 have an 8% probability of being toxic, the mean PEL quotients of 0.11–1.5 have a 21% probability of being toxic, the mean PEL quotients of 1.51–2.3 have a 49% probability of being toxic, and the mean PEL quotients of >2.3 have a 73% probability of being toxic [15].

### Statistical Analysis

Statistical methods were applied to process the analytical data in terms of the distribution and correlation among the studied parameters. Pearson's correlation coefficient analysis was performed to identify the relationship among heavy metals in sediments and their possible sources. The principal component analysis (PCA) of the normalized variables (Z-scores) was performed to extract significant principal components (PCs) and further reduce the contribution of variables with minor significance. After that factor analysis (FA) was conducted. These PCs were then subjected to varimax rotation to generate varifactors (VFs). The commercial statistics software package SPSS (version 19.0) for Windows was used for statistical analyses mentioned above in the present study.

The agglomerative hierarchical clustering (AHC) analysis was conducted on the normalized data set using Ward's method with Euclidean distances as a measure of similarity to assess the interrelationships among the sampling sites. The XLSTAT software (version 2013) was used in the AHC analysis.

## Results and Discussion

### Metals in total concentrations

**Figure 2:**
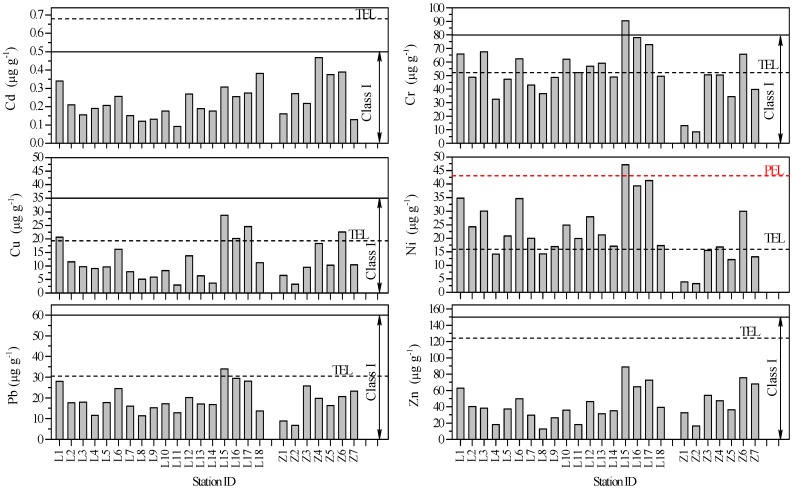
Information of the total concentrations of the studied metals. The spatial variations of studied metals in total concentrations of the surface sediments from the Laizhou Bay and the coastal Zhangzi Island. The horizontal dash lines represent their corresponding TEL or PEL concentrations; the horizontal solid lines represent their corresponding higher boundary values of Class I sediment category of China.

The spatial distribution of heavy metals is shown in Fig. 2 and the related information is summarized in Table 2. Based on the mean concentrations, the target elements in the surface sediments of the Laizhou Bay exhibited the following descending order: Cr (56.7 μg g^−1^) >Zn (41.5 μg g^−1^) > Ni (25.9 μg g^−1^) > Pb (19.4 μg g^−1^) > Cu (12.0 μg g^−1^) > Cd (0.22 μg g^−1^); in the Zhangzi Island the corresponding result was Zn (47.1 μg g^−1^) > Cr (37.4 μg g^−1^) > Pb (17.3 μg g^−1^) > Ni (13.5 μg g^−1^) > Cu (11.5 μg g^−1^) > Cd (0.29 μg g^−1^).

**Table 2:** Heavy metal concentrations in the surface sediments of the Laizhou Bay and the coastal Zhangzi Island; and the related values reported for the surface sediments of other marine areas of China are shown for comparison.

Location	Sampling date		Cd	Cr	Cu	Ni	Pb	Zn	References
Laizhou Bay, China	Oct., 2011	Range	0.09–0.38	32.4–90.4	2.9–28.7	14.1–47.1	11.4–34.0	12.8–88.6	Present study
		Mean	0.22	56.7	12.0	25.9	19.4	41.5	
Coastal Zhangzi Island, China	Nov., 2011	Range	0.13–0.47	8.4–65.6	3.3–22.5	3.2–30.0	6.7–25.8	16.2–75.4	Present study
		Mean	0.29	37.4	11.5	13.5	17.3	47.1	
Coastal Shandong Peninsula (Yellow Sea), China	2007		na^a^	57.8	20.0	31.2	28.4	74.7	[36]
Liaodong Bay, China	2009		na	46.4	19.4	22.5	31.8	71.7	[37]
Coastal East China Sea, China	May, 2009		0.30	84.2	33.1	36.1	28.0	102.4	[38]
Coastal Bohai Bay, China	May, 2008		0.22	101.4	38.5	40.7	34.7	131.1	[3]
Intertidal Bohai Bay, China	May, 2008		0.12	68.6	24.0	28.0	25.6	73.0	[39]
Jinzhou Bay, China	Oct., 2009		26.8	na	74.1	43.5	124.0	689.4	[14]
Laizhou Bay, China	May, 2007		0.081	57.1	13.3	19.4	20.2	59.4	[40]
Laizhou Bay, China	May, 2008		0.11	na	15.0	na	11.7	50.8	[41]
Daya Bay, China	Jan., 2006		0.052	na	20.8	31.2	45.7	113	[22]
North Bohai and Yellow Sea, China	Oct., 2008		0.15	47	13	na	25	60	[42]
Changjiang Estuary, China	Apr. and Aug., 2009		0.26	78.9	30.7	31.8	27.3	94.3	[43]
The Atlantic and Cantabric coasts, Spain	2001–2007		na	na	115.0	na	91.0	230.0	[44]
İzmit Bay, Turkey	Apr. 2002		5.10	75.0	66.4	41.2	104.7	961	[45]
Masan Bay, Korea	2004–2005		1.24	67.1	43.4	28.8	44.0	206.3	[46]
Gironde Estuary, France			0.48	78.4	24.5	31.7	46.8	168.0	[47]
San Francisco Bay, USA	Mar. 2000– Mar. 2001		0.14	19	33	33	19	60	[48]
Jade Bay, Germany	2009–2010		0.25	49	7	10	16	43	[49]

a na: not available.

Content unit is μg g^−1^ dry weight for all elements.

In the Laizhou Bay, the highest concentrations of Cr (90.4 μg g^−1^), Cu (28.7 μg g^−1^), Ni (47.1 μg g^−1^), Pb (30.4 μg g^−1^) and Zn (88.6 μg g^−1^) were all found in the surface sediments of site L15, which was about 10 km from the estuary of the Guanglihe River, Dongying City; the highest concentration of Cd (0.38 μg g^−1^) was found at site L18 which was about 8 km from the estuary of the Xiaoqinghe River, Weifang City. Relatively higher concentrations of all the six metals studied were also found at sites L1, L6, L16 and L17. L1 and L6 were about 10 km from the new and old mouths of the Yellow River, respectively, indicating the contribution to heavy metal content of terrigenous input. The samples from L17 and L16 had the first and the second highest percentages of fine fractions (clay and silt) which demonstrated that the deposition of fine grained materials physically controls the abundance and distribution of metals in sediments [16]. The information about the grain size and TOC in the sediments of this study has been described in detail in Gao et al. [35].

In the Zhangzi Island, the highest concentrations of Cr (62.2 μg g^−1^), Cu (22.5 μg g^−1^), Ni (30.0 μg g^−1^), Pb (25.8 μg g^−1^) and Zn (75.4 μg g^−1^) were all found in the surface sediments of site Z6, which was about 0.25 km from the coast and had the highest percentage of fine fractions and TOC [35]. The highest concentration of Cd (0.47 μg g^−1^) was found in the surface sediments of site Z4 located in the mariculture area about 0.25 km from the coast where sea cucumbers were farmed. The concentrations of the rest of the metals studied at site Z4 were also relatively higher than the other sites in the Zhangzi Island except Z6, which reflected the anthropogenic influence (e.g. fishing operations) on heavy metals.

For the comparison purpose, the average UCC values (Table 1) and related values reported about the surface sediments of some of the marine areas in China and other countries were also shown (Table 2). In the surface sediments from the Laizhou Bay, the mean total contents of Cd, Cr and Ni were clearly higher with respect to their corresponding average values in the UCC; the mean total content of Cu was close to its corresponding average value in the UCC. In the Zhangzi Island, only the values of Cd and Cr were higher than their corresponding average values in the UCC. The average concentrations of Cd, Cr, Ni, Pb and Zn in the Laizhou Bay, Cd, Cu, Ni, Pb and Zn in the Zhangzi Island were within the range identified in the other marine areas listed in Table 2. The average concentrations of Cu in the Laizhou Bay and Cr in the Zhangzi Island were lower than all the values in the other studies listed. All the average concentrations of the studied metals in the two areas were far below the values of the Jinzhou Bay in China, the Atlantic and Cantabric coasts in Spain, the İzmit Bay in Turkey and the Masan Bay in Korea which were much heavily polluted coastal zones in the world [14], [44], [45], [46]. All the average concentrations of the studied metals in the Zhangzi Island were close to the values of the Jade Bay in Germany where sediment quality was in good condition [49].

Correlation analyses have been widely used in environmental studies. They provide an effective way of revealing the relationships between multiple variables and parameters by which the factors as well as sources of chemical components could be better understood [5], [13], [38], [50]. The correlation matrix for the parameters studied was shown in Table 3. All the metals were significantly correlated with each other in the surface sediments of the Laizhou Bay, suggesting a major common origin in sediments in this area. The wastewater discharged from industrial sources into the surrounding rivers which runs into the Laizhou Bay could be responsible for this [18], [19], [20]. It has been reported that the deposition of fine grained materials and organic matter physically controls the abundance and distribution of metals in sediments [51]. The concentrations of Cu, Ni, Pb, and Zn appeared to be influenced by both the sediment grain size composition and the amount of organic matter; the concentration of Cr appeared to be more influenced by the sediment grain size composition than by the amount of organic matter; the concentration of Cd appeared to be influenced by neither the sediment grain size composition nor the amount of organic matter, perhaps because it is a typical anthropogenic element.

**Table 3:** Pearson correlation matrix for the sediment components.

		Cd	Cr	Cu	Ni	Pb	Zn	%Clay	%Silt	%Sand	%TOC
Laizhou Bay	Cd	1	0.472^c^	0.736^a^	0.539^c^	0.602^b^	0.722^a^	0.458	0.216	−0.331	0.381
	*p*		0.048	0.000	0.021	0.008	0.001	0.056	0.390	0.180	0.118
	Cr		1	0.786^a^	0.932^a^	0.894^a^	0.883^a^	0.535^c^	0.394	−0.473^c^	0.252
	*p*			0.000	0.000	0.000	0.000	0.022	0.105	0.047	0.312
	Cu			1	0.917^a^	0.924^a^	0.949^a^	0.759^a^	0.608^b^	−0.700^a^	0.530^c^
	*p*				0.000	0.000	0.000	0.000	0.007	0.001	0.024
	Ni				1	0.962^a^	0.938^a^	0.746^a^	0.593^b^	−0.685^b^	0.488^c^
	*p*					0.000	0.000	0.000	0.010	0.002	0.040
	Pb					1	0.965^a^	0.735^a^	0.572^c^	−0.668^b^	0.489^c^
	*p*						0.000	0.001	0.013	0.002	0.040
	Zn						1	0.708^a^	0.526^c^	−0.628^b^	0.484^c^
	*p*							0.001	0.025	0.005	0.042
Coastal Zhangzi Island	Cd	1	0.456	0.653	0.501	0.072	0.036	0.716	0.783^c^	−0.774^c^	0.525
	*p*		0.303	0.112	0.252	0.878	0.939	0.070	0.037	0.041	0.226
	Cr		1	0.884^b^	0.953^a^	0.858^c^	0.865^c^	0.745	0.842^c^	−0.824^c^	0.849^c^
	*p*			0.008	0.001	0.014	0.012	0.055	0.018	0.023	0.016
	Cu			1	0.928^b^	0.558	0.747	0.880^b^	0.929^b^	−0.926^b^	0.829^c^
	*p*				0.003	0.193	0.054	0.009	0.003	0.003	0.021
	Ni				1	0.694	0.848^c^	0.884^b^	0.901^b^	−0.906^b^	0.932^b^
	*p*					0.083	0.016	0.008	0.006	0.005	0.002
	Pb					1	0.830^c^	0.318	0.458	−0.424	0.522
	*p*						0.021	0.487	0.302	0.343	0.229
	Zn						1	0.550	0.565	−0.567	0.667
	*p*							0.201	0.187	0.184	0.101

a *p*<0.001.

b 0.001<*p*<0.01.

c 0.01<*p*<0.05.

n = 18 for the Laizhou Bay and n = 7 for the coastal Zhangzi Island.

In the surface sediments of the Zhangzi Island, Cr was significantly correlated with the other studied metals except Cd, suggesting a wide origin of Cr in sediments of this area. The concentrations of Cr, Cu and Ni appeared to be influenced by both the sediment grain size composition and the amount of organic matter, and the concentration of Cd appeared to be influenced only by grain size composition. The concentrations of Pb and Zn were significantly correlated, whereas both of them had no significant correlation with grain size composition and the amount of organic matter, which indicated that they had a major common origin in sediments but not the same as the other metals. Zn might be released from the anti-corrosion paints used on ship hulls or from other anthropogenic sources [52], [53], and Pb might be released with engine exhaust of ships [54], [55], [56].

### Metal fractionation

**Figure 3:**
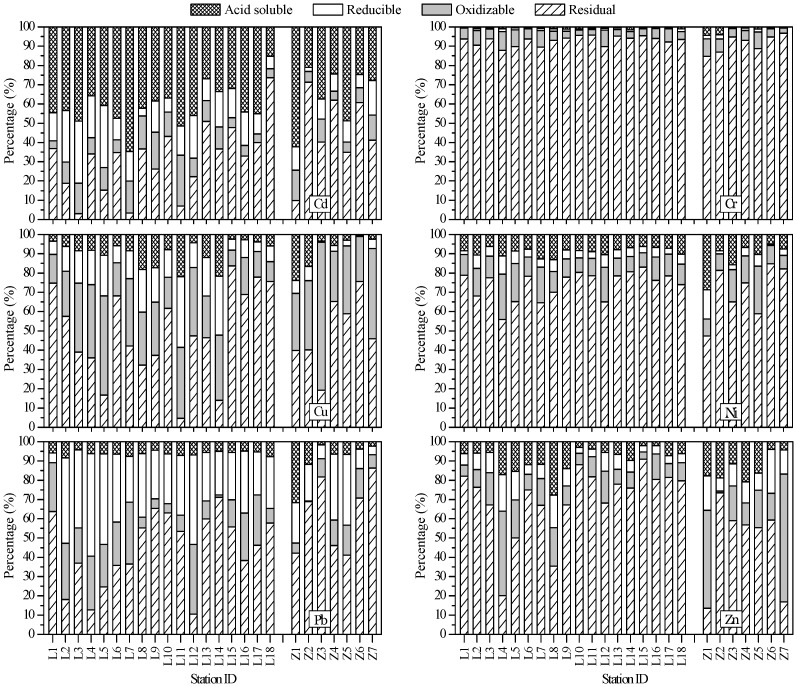
Geochemical phases of the studied metals. The distributions of studied metals in different geochemical phases of the surface sediments from the Laizhou Bay and the coastal Zhangzi Island.

The sequential extraction technique is proposed to provide information about the strength and ways of metals associating with sediments and thus predict the possible metal impact on biota in aquatic ecosystems [16], [57]. The metals in acid soluble fraction (i.e., the exchangeable and bound to carbonate fractions) are mainly introduced by human activities and are considered to be weakly bound. This fraction may equilibrate with aqueous phase and thus become more rapidly bioavailable and cause environmental toxicity [58]. The reducible fraction (bound to Fe/Mn oxyhydroxides) and the oxidizable fraction (bound to organic matter) can be mobilized when environmental conditions become increasingly reducing or oxidizing, respectively [58]. The detrital fraction which is composed of metals present in the inert fraction, being of lattice origin or primary mineral phases, can be regarded as a measure of contribution by natural sources [59]. The percentages of heavy metal concentrations that were extracted in each step of the sequential extraction procedure used in this study were presented in Fig. 3, and the pearson correlation matrix for metal fractionations with grain size and TOC was shown in Table 4.

**Table 4:** Pearson correlation matrix for metal fractionations with grain size and TOC.

		Laizhou Bay				Coastal Zhangzi Island			
		F1	F2	F3	F4	F1	F2	F3	F4
Cd	%Clay	0.435	0.344	−0.516^c^	−0.235	−0.266	−0.288	−0.441	0.356
	*p*	0.071	0.163	0.028	0.347	0.564	0.531	0.322	0.433
	%Silt	0.381	0.452	−0.450	−0.275	−0.323	−0.277	−0.463	0.400
	*p*	0.119	0.060	0.061	0.270	0.480	0.548	0.295	0.374
	%Sand	−0.421	−0.424	0.498^c^	0.270	0.311	0.283	0.462	−0.392
	*p*	0.082	0.080	0.035	0.279	0.498	0.539	0.296	0.384
	%TOC	0.398	0.501^c^	−0.403	−0.323	−0.328	−0.272	−0.241	0.355
	*p*	0.102	0.034	0.097	0.191	0.473	0.556	0.603	0.434
Cr	%Clay	−0.673^b^	−0.511^c^	0.525^c^	−0.324	−0.484	−0.307	−0.184	0.338
	*p*	0.002	0.030	0.025	0.190	0.271	0.504	0.693	0.458
	%Silt	−0.560^c^	−0.343	0.600^b^	−0.436	−0.586	−0.385	−0.276	0.439
	*p*	0.016	0.164	0.009	0.070	0.167	0.394	0.550	0.324
	%Sand	0.634^b^	0.432	−0.593^b^	0.406	0.565	0.367	0.253	−0.416
	*p*	0.005	0.074	0.010	0.095	0.187	0.418	0.584	0.353
	%TOC	−0.566^c^	−0.297	0.671^b^	−0.511^c^	−0.544	−0.561	−0.399	0.520
	*p*	0.014	0.231	0.002	0.030	0.207	0.190	0.375	0.232
Cu	%Clay	−0.785^a^	−0.651^b^	−0.192	0.542^c^	−0.559	−0.701	−0.429	0.781^c^
	*p*	0.000	0.003	0.445	0.020	0.192	0.079	0.336	0.038
	%Silt	−0.695^a^	−0.535^c^	0.023	0.369	−0.598	−0.800^c^	−0.296	0.684
	*p*	0.001	0.022	0.927	0.132	0.156	0.031	0.519	0.090
	%Sand	0.764^a^	0.609^b^	0.070	−0.461	0.594	0.781^c^	0.337	−0.719
	*p*	0.000	0.007	0.782	0.054	0.160	0.038	0.460	0.069
	%TOC	−0.636^b^	−0.492^a^	0.060	0.319	−0.597	−0.853^c^	−0.070	0.472
	*p*	0.005	0.038	0.813	0.197	0.157	0.015	0.881	0.285
Ni	%Clay	−0.149	−0.131	0.290	−0.108	−0.485	−0.385	0.186	0.335
	*p*	0.556	0.604	0.244	0.671	0.270	0.394	0.689	0.463
	%Silt	−0.169	0.144	0.448	−0.266	−0.475	−0.414	0.237	0.316
	*p*	0.501	0.569	0.062	0.285	0.281	0.356	0.608	0.489
	%Sand	0.168	−0.029	−0.398	0.208	0.483	0.410	−0.226	−0.325
	*p*	0.506	0.908	0.102	0.407	0.272	0.361	0.626	0.477
	%TOC	0.025	0.106	0.445	−0.310	−0.400	−0.500	0.095	0.366
	*p*	0.923	0.676	0.064	0.210	0.374	0.254	0.840	0.420
Pb	%Clay	0.083	0.011	0.839^a^	−0.496^c^	−0.366	0.098	0.792^c^	−0.101
	*p*	0.744	0.965	0.000	0.036	0.420	0.834	0.034	0.830
	%Silt	−0.111	0.264	0.736^a^	−0.581^c^	−0.436	0.134	0.820^c^	−0.095
	*p*	0.661	0.289	0.000	0.012	0.329	0.774	0.024	0.839
	%Sand	0.031	−0.165	−0.813^a^	0.569^c^	0.421	−0.126	−0.822^c^	0.098
	*p*	0.903	0.514	0.000	0.014	0.347	0.788	0.023	0.835
	%TOC	0.271	0.258	0.841^a^	−0.660^b^	−0.470	−0.216	0.679	0.201
	*p*	0.277	0.301	0.000	0.003	0.287	0.642	0.093	0.666
Zn	%Clay	−0.410	−0.183	−0.112	0.249	−0.355	0.584	−0.415	0.392
	*p*	0.091	0.467	0.658	0.319	0.435	0.169	0.354	0.384
	%Silt	−0.189	0.119	0.216	−0.071	−0.236	0.493	−0.482	0.447
	*p*	0.453	0.639	0.390	0.780	0.611	0.261	0.273	0.315
	%Sand	0.294	0.009	**−**0.081	−0.066	0.271	−0.524	0.469	−0.437
	*p*	0.237	0.973	0.748	0.794	0.556	0.228	0.289	0.327
	%TOC	−0.204	0.035	−0.034	0.081	−0.482	0.613	−0.413	0.422
	*p*	0.418	0.891	0.894	0.751	0.273	0.144	0.357	0.346

a *p*<0.001.

b 0.001<*p*<0.01.

c 0.01<*p*<0.05.

n = 18 for the Laizhou Bay and n = 7 for the coastal Zhangzi Island; F1, F2, F3 and F4 represent acid soluble, reducible, oxidizable and residual fraction, respectively.

Each studied metal displayed the similar compositional characteristics between the surface sediments in the Laizhou Bay and the Zhangzi Island. On average, the residual fraction was the most dominant one for all the studied metals except Cd, indicating the paramount mineralogical origin of these metals; while differences among sampling sites were obvious, which might result from the combined effects of the physicochemical conditions of the sedimentary environment, the intensity of human activities and so on (Fig. 3).

Generally, except for Cd, the relative proportions of metals in the acid soluble fraction were very low, especially for Cr (≈1% of total concentration). In the Laizhou Bay, the very low concentrations of Cr and Cu in the acid soluble fraction could still be partly from mineral sources, because significant positive correlation between this fraction and sand was observed (Table 4). Only Cd had observable contents of the acid soluble fraction with the mean values of 41.0% and 35.2% in the Laizhou Bay and the Zhangzi Island, respectively. The result was similar to the previous study carried out in the Bohai Bay by Gao and Chen [3]. The excessive input of Cd into water induced by phosphorus fertilizer has been widely reported [60], [61]. In addition, the presence of Cd could also be a result of road traffic, which has been described as an important source of Cd emission [62].

Pb exhibited the highest proportion within the reducible fraction (5.0–53.2% in the Laizhou Bay; 4.4–36.7% in the Zhangzi Island) among the six studied metals, which might be the result of the higher stability of Pb-oxides, and also could be attributed to the adsorption, flocculation and co-precipitation of heavy metals with the colloids of Fe and Mn oxyhydroxide [63]. The same result was also reported by other researchers [3], [16], [22], [39]. In both of the studied areas, Pb in oxidizable fraction was significantly correlated with clay and silt, indicating this fraction might be mainly from terrestrial source (Table 4).

On average, the proportions of non-residual Cu, Pb and Zn were identified being the highest in the oxidizble fraction in the Laizhou Bay; in the Zhangzi Island, the proportions of non-residual Cu, Zn and Ni were the highest in the oxidizble fraction. In both of the two studied areas, Cu had the highest proportion (8.2–51.5% in the Laizhou Bay; 23.4–76.8% in the Zhangzi Island) among these metals. This result could be explained by the affinity of metals with organic matter, especially humic substances, which are both the components of natural organic matter and chemical actives in complexing metals [64], [65]. The partitioning patterns of Zn and Cu were somewhat very similar with each other. The observed non-residual fractions of Zn and Cu might be the result of ZnO and Cu_2_O released from the anti-corrosion paints used on ship hulls during the maintenance of ships or from other anthropogenic sources [52], [53], [66]. The residual fractions of Cu in both the Laizhou Bay and the Zhangzi Island could be from rock weathering source because significant positive correlation between this fraction and clay was observed (Table 4).

The concentration of Cr showed a completely different pattern from the others. Among the non-residual fraction, Cr had the highest content in oxidizable fraction (2.8–9.7% in the Laizhou Bay; 2.6–9.1% in the Zhangzi Island). In the Laizhou Bay, Cr in oxidizable fraction was significantly correlated with clay, silt and TOC, indicating this fraction was mainly from terrestrial source and was influenced by the amount of organic matter (Table 4). Substantial amounts of Cr were found in the residual phases (87.8–95.7% in the Laizhou Bay; 84.8–96.8% in the Zhangzi Island). This suggested that Cr had the strongest associations with the crystalline sedimentary components.

Pearson correlation analysis can partly give information on the sources of the metals in the environment. To explore this topic further, risk indices, principal component analysis and factor analysis were used later in this article.

### Risk assessment of heavy metals

#### 1. Risk assessment according to SQGs

The higher boundary values of Class I sediment category of China [9] and the corresponding TEL and PEL concentrations were listed in Table 1 and marked in Fig. 2. All the concentrations of metals at all the sites in both the Laizhou Bay and the Zhangzi Island were below the values of the upper limit for Class I sediment except for Cr in the surface sediments of site L15. This indicated that the sedimentary environments of both the Laizhou Bay and the Zhangzi Island were in good condition according to the marine sediment quality of China.

**Figure 4:**
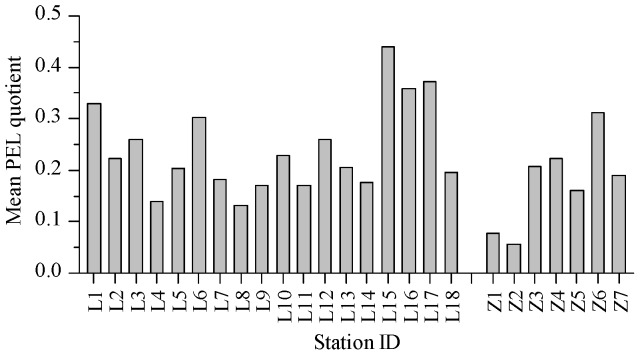
The spatial distribution of mean PEL quotient values. The histogram shows spatial distribution of mean PEL quotient values in the surface sediments of the Laizhou Bay and the coastal Zhangzi Island.

The data of this study also suggested that no site exceeded the TEL guideline for Cd or Zn in both the Laizhou Bay and the Zhangzi Island. In the case of other metals in the Laizhou Bay, 50%, 78%, 11% and 94% of sites were below the TEL guideline for Cr, Cu, Ni and Pb, respectively; site L15 was even above the PEL guideline for Ni. In the case of other metals in the Zhangzi Island, site Z6 was above the TEL guideline for Cr and Cu; sites Z4 and Z6 were above the TEL guideline for Ni; no site exceeded the TEL guideline for Pb. As shown in Fig. 4, in the surface sediments of the Laizhou Bay and the Zhangzi Island, the combination of the six studied metals of sites Z1 and Z2 might have an 8% probability of being toxic, and these metals might have a 21% probability of being toxic for all the other sites.

**Figure 5:**
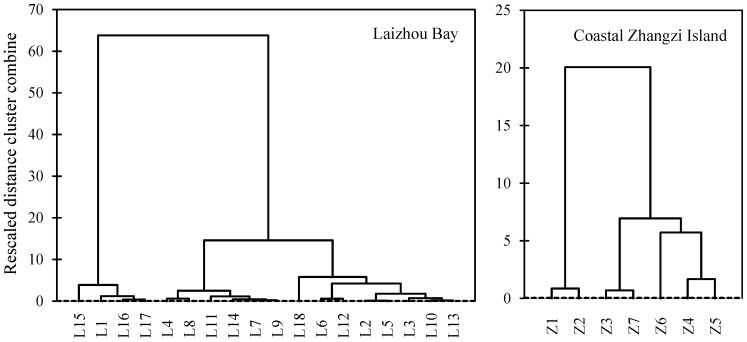
Dendrogram showing clustering of sampling sites in the two studied areas.

The result of the hierarchical cluster analysis of the sampling sites based on the data of metals in total concentrations and SQGs was shown in Fig. 5. Three main different clusters could be observed for the Laizhou Bay. Cluster 1 involved several sites (L1, L15, L16 and L17) near to the Yellow River mouths and in the southwestern Laizhou Bay, respectively, indicating the sites were moderately to heavily contaminated according to the SQGs; Cluster 2 was made up of the sites L4, L7, L8, L9, L11 and L14 which were uncontaminated or less contaminated; Cluster 3 was made up of the rest sites which were moderately contaminated. Two main different clusters could be observed for the Zhangzi Island. Cluster 1 included two sites (Z1 and Z2) in the intertidal zone which were uncontaminated according to the SQGs; Cluster 2 was made up of the rest sites which were moderately contaminated.

#### 2. Risk assessment according to contamination and ecological risk indices

**Figure 6:**
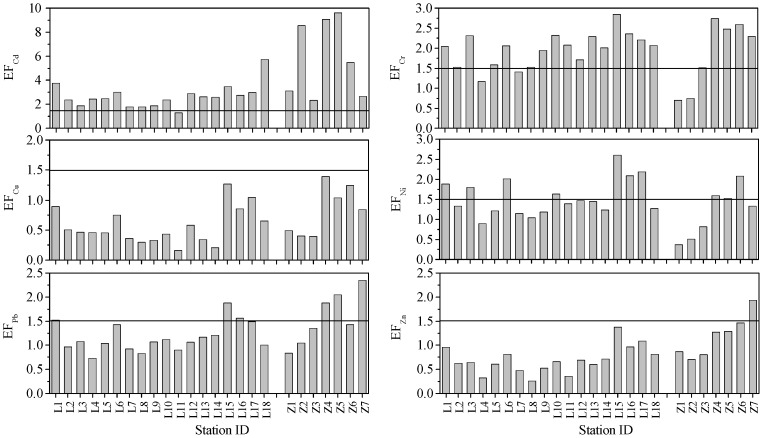
The spatial distributions of EF values. The histogram shows spatial distributions of EF values for heavy metals in the surface sediments of the Laizhou Bay and the coastal Zhangzi Island. The horizontal lines represent EF value of 1.5.

According to Zhang and Liu [67], EF values between 0.5 and 1.5 indicate that the given metal is entirely derived from crustal materials or natural weathering processes, whereas EF values higher than 1.5 suggest that a significant portion of metal is delivered from non-crustal materials and the sources are more likely to be anthropogenic. The spatial distributions of calculated EFs for each of the studied metals were shown in Fig. 6. The mean EF values of Cd and Cr suggested their enrichments in most surface sediments of the Laizhou Bay and the Zhangzi Island. In the Laizhou Bay, the highest EF value of Cd was recorded at site L18 (5.7) near the estuary of the Xiaoqinghe River; in the Zhangzi Island, the highest EF value of Cd was found at site Z5 (9.6) 1.4 km away from the coast where scallops were farmed; sites Z2, Z4 and Z6 in the Zhangzi Island also had high EF values of Cd within the range of 5 to 10. This indicated that Cd in the surface sediments of these five sites was in moderately severe enrichment [12]. The highest EF values of Cr were recorded at site L15 (2.8) near the Guanglihe River estuary in the Laizhou Bay and at site Z4 (2.7) where sea cucumbers were farmed in the Zhangzi Island. According to the EF values, the contribution from anthropogenic sources was negligible for Zn at all sites in the Laizhou Bay and for Cu in all sites of the Laizhou Bay and the Zhangzi Island. Generally, Ni and Pb were slightly enriched in the surface sediments near the Yellow River Estuary and the southwestern Laizhou Bay and sites Z4–Z7 of the Zhangzi Island; Zn was also slightly enriched at Z7 which was near a small wharf.

**Figure 7:**
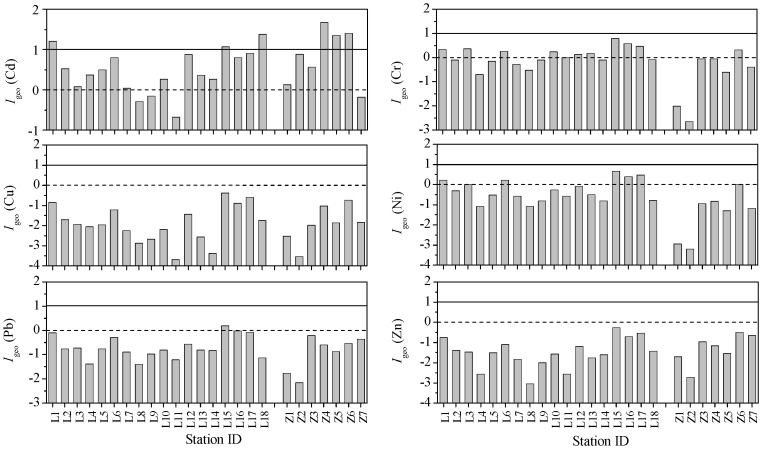
The spatial distributions of *I*_geo_ values. The histogram shows the spatial distributions of *I*_geo_ values for heavy metals in the surface sediments of the Laizhou Bay and the coastal Zhangzi Island. The horizontal dash and solid lines represent *I*_geo_ values of 0 and 1, respectively.

The spatial distributions of calculated *I*_geo_ values for each of the studied metals were shown in Fig. 7. Most of the *I*_geo_ values of Cd were between 0 and 1 which showed that these sites were uncontaminated to moderately contaminated. *I*_geo_ values of Cd in the surface sediments of the sites near to the mouths of the Yellow River, the Guanglihe River and the Xiaoqinghe River in the Laizhou Bay and all the three sites in the coastal waters of the Zhangzi Island were between 1 and 2, further indicating these sites were more affected by human activities than other sites. The *I*_geo_ values suggested that Cu and Zn at all sites in both the Laizhou Bay and the Zhangzi Island were in the uncontaminated level, and this was true for Pb except at site L15 where the *I*_geo_ value of Pb was a little higher than 0. The values of *I*_geo_ for Cr and Ni at most sites were <0 except at several ones that were near to the Yellow River mouths and in the southwestern Laizhou Bay; for all the sampling sites in the Zhangzi Island, the *I*_geo_ values indicated that their surface sediments were practically uncontaminated by Ni, and Cr presented the same situation like Ni except at site Z6 which had a condition of slight Cr pollution.

**Figure 8:**
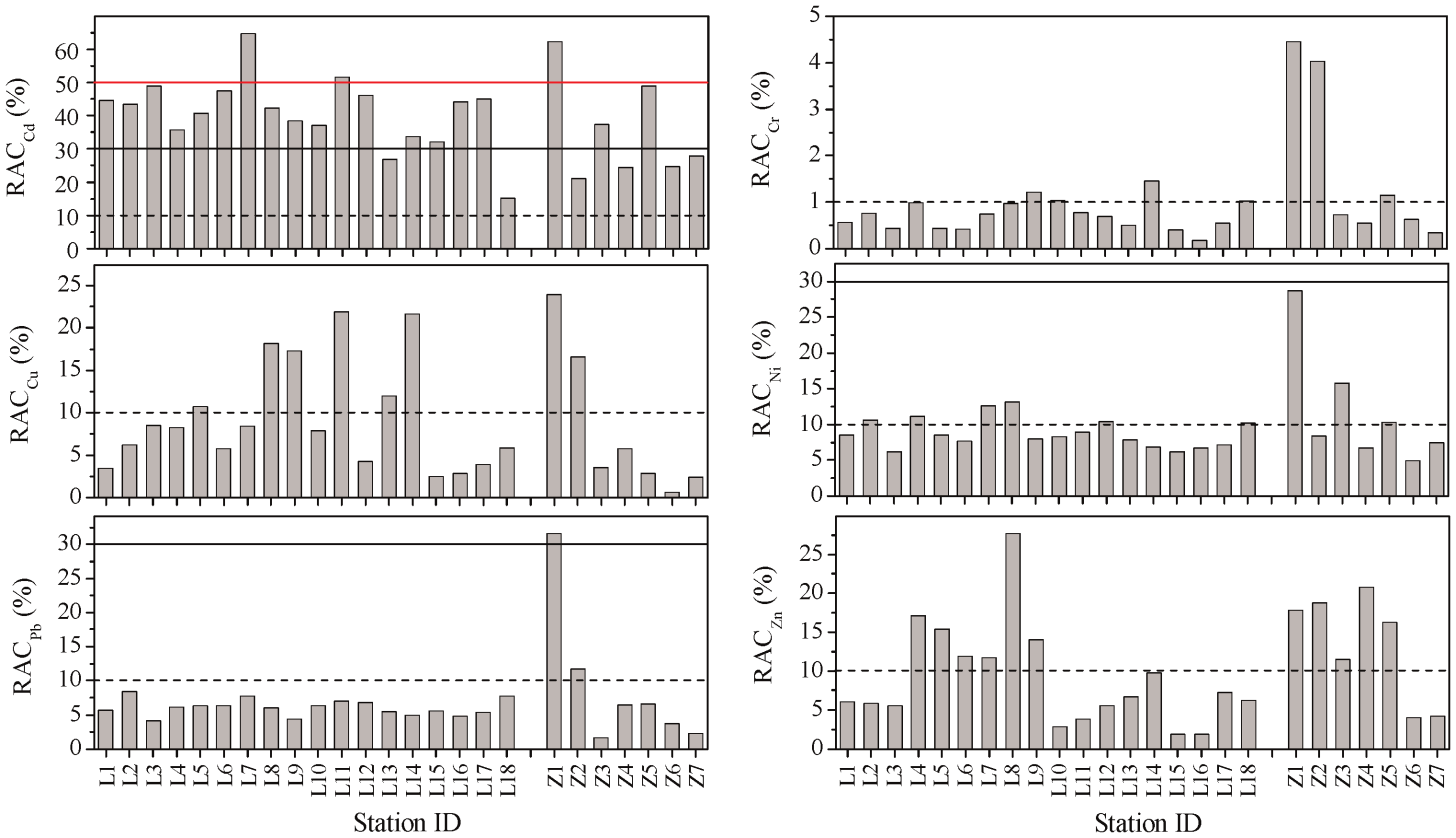
The spatial distributions of RAC values. The histogram shows the spatial distributions of RAC values (%) for heavy metals in the surface sediments of the Laizhou Bay and the coastal Zhangzi Island. The horizontal dash lines represent RAC values (%) of 1 or 10. The horizontal solid lines represent RAC values (%) of 30 or 50.

The spatial distributions of calculated RACs for each of the studied metals were shown in Fig. 8. It showed that most of the sites suffered high risks from Cd (30%<RAC≤50%). Risk from Cd in the surface sediments of sites L7 and L11 in the Laizhou Bay and site Z1 in the Zhangzi Island was very high (RAC>50%). Although the total concentration of Cd in the surface sediments of Z1 was very low, the proportion of acid soluble fraction of it was high; this might be because Z1 was near a seedling factory and was severely impacted by the discharge from that factory. The values of RAC at site Z1 were also higher than the other sites in the Zhangzi Island for Cu (24.0%), Ni (28.8%) and Pb (31.6%). Cr at all the sites had no to low risk; Cu, Ni and Zn at most sites had low to medium risk; Pb at all the sites had low risk except at sites Z1 and Z2 which had high risk and medium risk, respectively.

**Figure 9:**
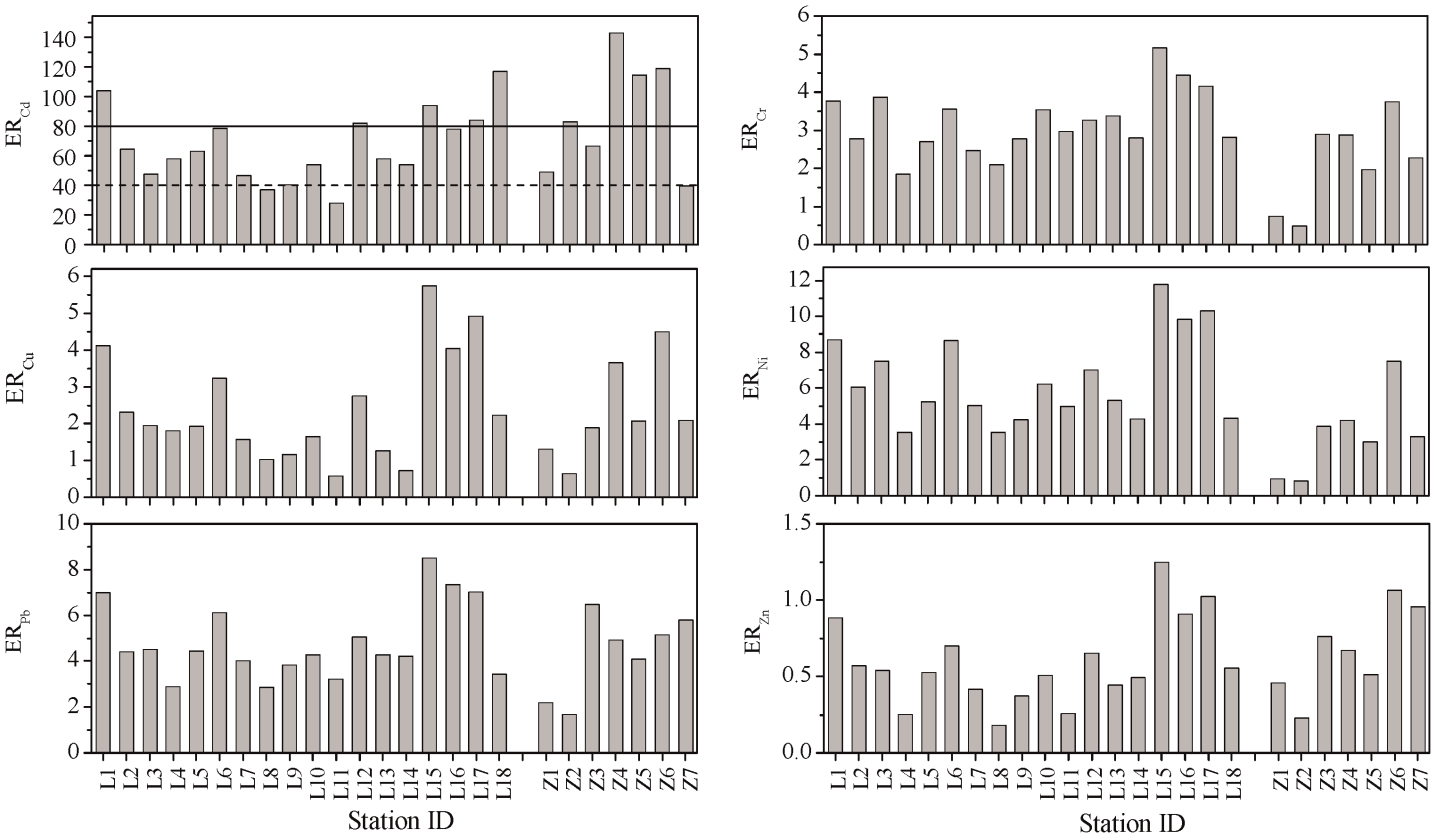
The spatial distributions of ER values. The histogram shows the spatial distributions of ER values for heavy metals in the surface sediments of the Laizhou Bay and the coastal Zhangzi Island. The horizontal dash and solid lines represent ER values of 40 and 80, respectively.

The spatial distributions of calculated ERs for each of the studied metals were shown in Fig. 9. According to ER index, potential ecological risk posed by Cr, Cu, Ni, Pb and Zn was very low at all the sites. However, the potential ecological risk posed by Cd was obvious at most of the sites. Except for sites L8 and L11 in the Laizhou Bay and Z7 in the Zhangzi Island, all the other sites at least suffered moderate risk from Cd (40<ER≤80). Sites L1, L15, L17 and L18 in the Laizhou Bay and sites Z4–Z6 suffered considerable risk from Cd (80<ER≤160). The sites which suffered considerable risk from Cd according to ER index were also moderately contaminated according to *I*_geo_ index.

**Figure 10:**
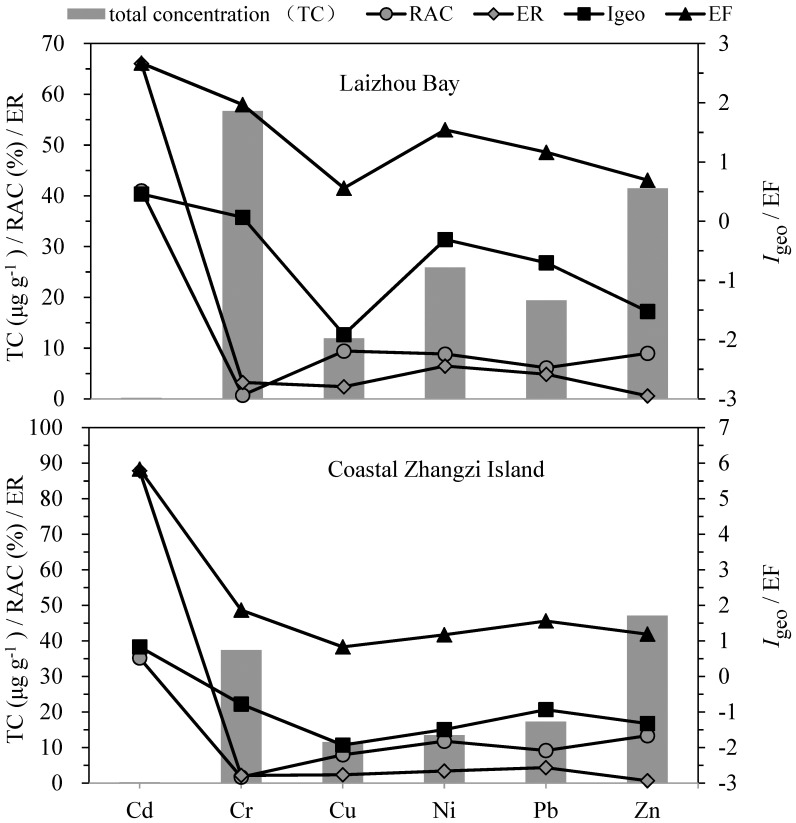
The mean values of EF, *I*_geo_, RAC, ER and total concentration for metals.

The mean values of EF, *I*_geo_, RAC and ER of the six studied metals and their total concentrations were summarized in Fig. 10. It showed clearly that Cd had the highest potential risk according to all the four indices. However, inconsistent conclusions for the other five metals according to these four indices could be drawn. In addition, the results of EF and *I*_geo_ were consistent with the trend of the total metal concentrations except for Cd; meanwhile the results of RAC and ER had no consistency with the trend of their corresponding total metal concentrations. The explanation for this might be as the following: i) the EF and *I*_geo_ indices are calculated mainly based on the concentrations of the total metals and enrichment levels. ii) The ER index is based on the toxic-response factor besides the total concentration. For example, though total concentration of Cd is usually pretty lower than Zn in sediments, but the higher value of toxic-response factor of Cd (Tr = 30) might make it much more toxic than Zn whose toxic-response factor is only 1. iii) The RAC index, which reflects the potential mobility of sedimentary metals, is based on the chemical form of a given metal which has no direct relationship with its total concentration. It is generally accepted that the fractionation can give more information on the bio-availability and bio-toxicity of a certain metal than the total concentration [3], [16], [38], [68]. However, the results of the present study indicated the necessity of further verifying the prediction accuracy of the fractionation based methods.

It is generally known that the mechanisms of these SQS and indices are divergent and they are used to assess the environmental risk from different angles. Neither the total concentration based index nor the chemical fractionation based index alone could be sufficient in revealing the biogeochemical information of heavy metals in sediments. However, the trend of risk level according to the assessment results of them should be consistent or at least should not be opposite to each other; if not, misleading results of risk assessment may even cause misjudgment during the formulation and enforcement of public policies by government. Therefore, the merits and defects should be evaluated before the application of these assessment methods, and it is necessary to use multiple evaluation methods. Different metals may have different risk gradation ranges. For instance, the differences between metal fractionation characteristics of Cd and Cr are often substantial, but the risks of these two metals ranked by RAC indexes are within the same gradation range that this may not be true. We believe that a more comprehensive index to reveal the information of the concentration, the chemical fractionation and the toxic-response factor of heavy metals should be developed. Therefore, we suggest that more toxicity tests, benthic community analyses, bio-accumulation tests, and a combination of these should be carried out to make the risk assessment of sedimentary heavy metals more accurate and reliable.

### Principal component analysis/factor analysis

**Figure 11:**
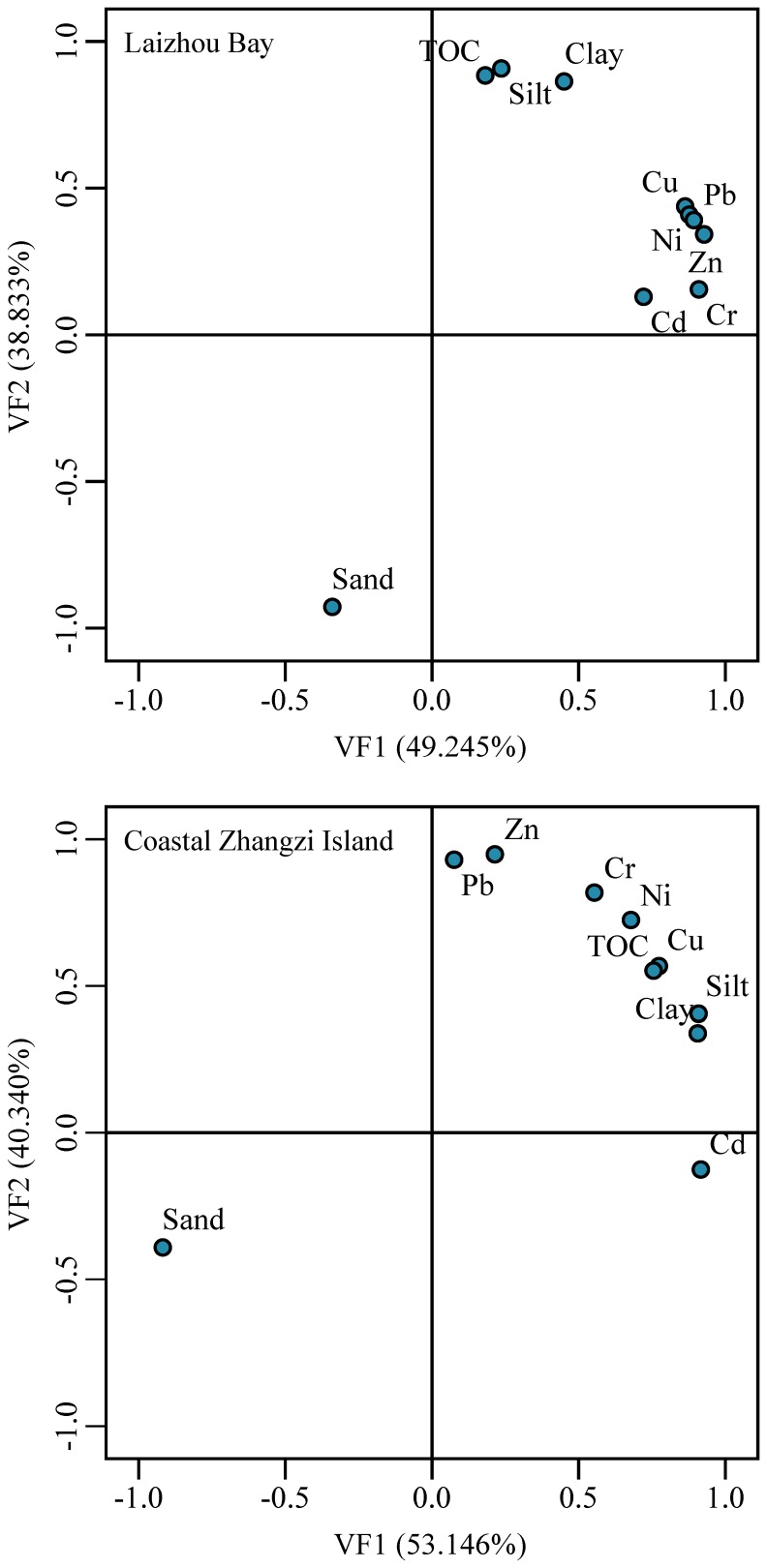
Loading plots of the principal components obtained for the data set.

PCA/FA was performed to identify interrelationships of the six studied heavy metals and the major constituents of the sediments (TOC, clay, silt and sand). Table 5 showed that there were two PCs for the surface sediments in both the Laizhou Bay and the Zhangzi Island. The loading plots of the VFs were presented in Fig. 11. These PCs were the ones with eigenvalues larger than 1, and altogether they accounted for 88.1% and 93.5% of the variance in the data of the Laizhou Bay and the Zhangzi Island, respectively.

**Table 5:** Loadings of experimental variables on significant principal components for the data sets of the Laizhou Bay and the coastal Zhangzi Island.

	Laizhou Bay		Coastal Zhangzi Island	
	PC1	PC2	PC1	PC2
Cd	**0.639**	0.359	**0.916**	**−**0.126
Cr	**0.800**	0.460	**0.553**	**0.818**
Cu	**0.944**	0.213	**0.774**	**0.567**
Ni	**0.937**	0.243	**0.678**	**0.725**
Pb	**0.937**	0.268	0.075	**0.930**
Zn	**0.933**	0.327	0.214	**0.949**
Clay	**0.897**	**−**0.379	**0.906**	0.339
Silt	**0.760**	**−0.549**	**0.909**	0.405
Sand	**−0.853**	**0.498**	**−0.918**	**−**0.391
TOC	**0.703**	**−0.566**	**0.755**	**0.552**
Eigenvalue	4.925	3.883	5.315	4.034
% Total variance	49.245	38.833	53.146	40.340
Cumulative % variance	49.245	88.078	53.146	93.486

Bold values indicate strong loadings.

In the Laizhou Bay, PC1 which explained 49.3% of the total variance was positively related to all the heavy metals and major constituents except sand. Sand was significantly negatively related to PC1. The high loading of clay, silt and TOC with PC1 highlighted the influence of fine grained minerals and organic matter on the distributions of heavy metals in the sediments of the Laizhou Bay, and revealed that these metals were mainly from terrestrial source especially via rivers [68]. PC2 which explained 38.8% of the total variance was only positively related to sand, indicating that sand could hardly capture metal ions.

In the Zhangzi Island, PC1 was positively related to Cd, Cr, Cu, Ni, Clay, Silt and TOC. This PC represented terrestrial sources. PC2 was also positively related to Cr, Cu, Ni and TOC, indicating they were from both terrestrial inputs and biogenic sources [68]. Zn and Pb were only positively related to PC2, indicating their different sources with the other studied metals. Zn and Pb might be released from ships and from biogenic sources. In addition, previous studies have shown that a large amount of Pb is supplied by the precipitation of aerosols in coastal environments [69]. So the precipitation of aerosols might be another important source of Pb in the surface sediments of the Zhangzi Island.

## Conclusions

This study investigated the total concentrations and fractionation of heavy metals in the surface sediments from the Laizhou Bay and the Zhangzi Island. The relatively higher concentrations of metals in the Laizhou Bay were mainly distributed near to the new and old mouths of the Yellow River, the mouths of Guanglihe and Xiaoqinghe Rivers, and in the middle of the Bay. The relatively higher concentrations of metals in the Zhangzi Island were mainly distributed in and near the mariculture areas. In the Laizhou Bay, all the metals studied were mainly from terrestrial sources, and especially Cd, Cr and Ni had obvious anthropogenic sources; in the Zhangzi Island, both natural and anthropogenic sources contributed significantly to the metal contents.

The marine sediment quality of China showed that the sedimentary environment in both the Laizhou Bay and the Zhangzi Island were in good condition. TEL/PEL guidelines revealed that adverse biological effects might occur frequently in some areas in the Laizhou Bay and the Zhangzi Island especially from Cr and Ni. Based on the mean PEL quotient, surface sediments of sites Z1 and Z2 had an 8% probability of toxicity, and surface sediments of the rest sites of the two studied areas had a 21% probability of toxicity.

All the four risk assessment indices used in this study revealed an obvious pollution risk by Cd, especially in sites near the river mouths and in the southwestern Laizhou Bay and in the coastal waters of the Zhangzi Island. Nevertheless, contradictory conclusions could be obtained when different indices and SQGs are used. Significant negative correlations between RAC and the other indices and between RAC and the total metal concentration for Cd, Cr, Cu and Ni were found. We suggest that toxicity tests, bio-accumulation tests, and other related experiments should be further carried out in order to make the risk assessment methods more accurate and reliable in the analysis of sedimentary heavy metals.
